# Associations between echocardiographic traits and artificial intelligence-enabled electrocardiography predictions of heart failure

**DOI:** 10.1093/ehjdh/ztag141

**Published:** 2026-09-11

**Authors:** Elias Stenhede, Eivind Bjørkan Orstad, Torbjørn Omland, Henrik Schirmer, Arian Ranjbar

**Affiliations:** Medical Technology & E-Health, Akershus University Hospital, Sykehusveien 25, 1478 Lørenskog, Norway; Faculty of Medicine, University of Oslo, Postboks 1078 Blindern, 0372 Oslo, Norway; Faculty of Medicine, University of Oslo, Postboks 1078 Blindern, 0372 Oslo, Norway; Department of Cardiology, Akershus University Hospital, 1378 Lørenskog, Norway; Department of Cardiology, Akershus University Hospital, 1378 Lørenskog, Norway; Institute of Clinical Medicine, Campus Ahus, University of Oslo, Oslo, Norway; Department of Cardiology, Akershus University Hospital, 1378 Lørenskog, Norway; Institute of Clinical Medicine, Campus Ahus, University of Oslo, Oslo, Norway; Medical Technology & E-Health, Akershus University Hospital, Sykehusveien 25, 1478 Lørenskog, Norway

**Keywords:** Heart failure, Global longitudinal strain, AI, Electrocardiogram, Echocardiogram

## Abstract

**Aims:**

Artificial intelligence-enabled electrocardiography (AI–ECG) can detect heart failure (HF), including disease not captured by left ventricular ejection fraction (LVEF), but the cardiac phenotypes underlying AI–ECG HF prediction remain unclear. We therefore investigated whether an AI–ECG HF prediction score aligns with established echocardiographic measures of myocardial dysfunction, remodelling, and filling pressures.

**Methods and results:**

We retrospectively analysed ECG and echocardiography data from 8147 patients who underwent both examinations within 3 days at Akershus University Hospital between 1 January 2023 and 1 June 2025. A previously developed AI–ECG model, pragmatically trained using ICD-10 HF codes and N-terminal pro-B-type natriuretic peptide thresholds, was applied to all electrocardiograms. Spearman’s rank correlation *ρ* quantified associations between echocardiographic parameters and the AI–ECG HF prediction score. Subgroup analyses were performed by sex and LVEF. External replication of shared echocardiographic associations included 36 286 ECG–echocardiography pairs from Columbia University Irving Medical Center. Global longitudinal strain (GLS) showed the strongest correlation (*ρ* = 0.57), followed by mitral annular plane systolic excursion (MAPSE; *ρ* = −0.49) and LVEF (*ρ* = −0.45). In patients with LVEF ≥50%, correlations remained substantial for GLS, MAPSE, and diastolic-related parameters. Volumetric left ventricular indices correlated less strongly in women, whereas diastolic indices showed stronger correlations in women than in men.

**Conclusion:**

Echocardiographic trait characterization showed that the AI–ECG HF prediction score aligned primarily with measures of systolic function, particularly GLS, while also being associated with diastolic-related abnormalities in patients with preserved LVEF. This approach may inform future studies of model interpretability and refinement.

## Graphical Abstract

**Graphical Abstract:**
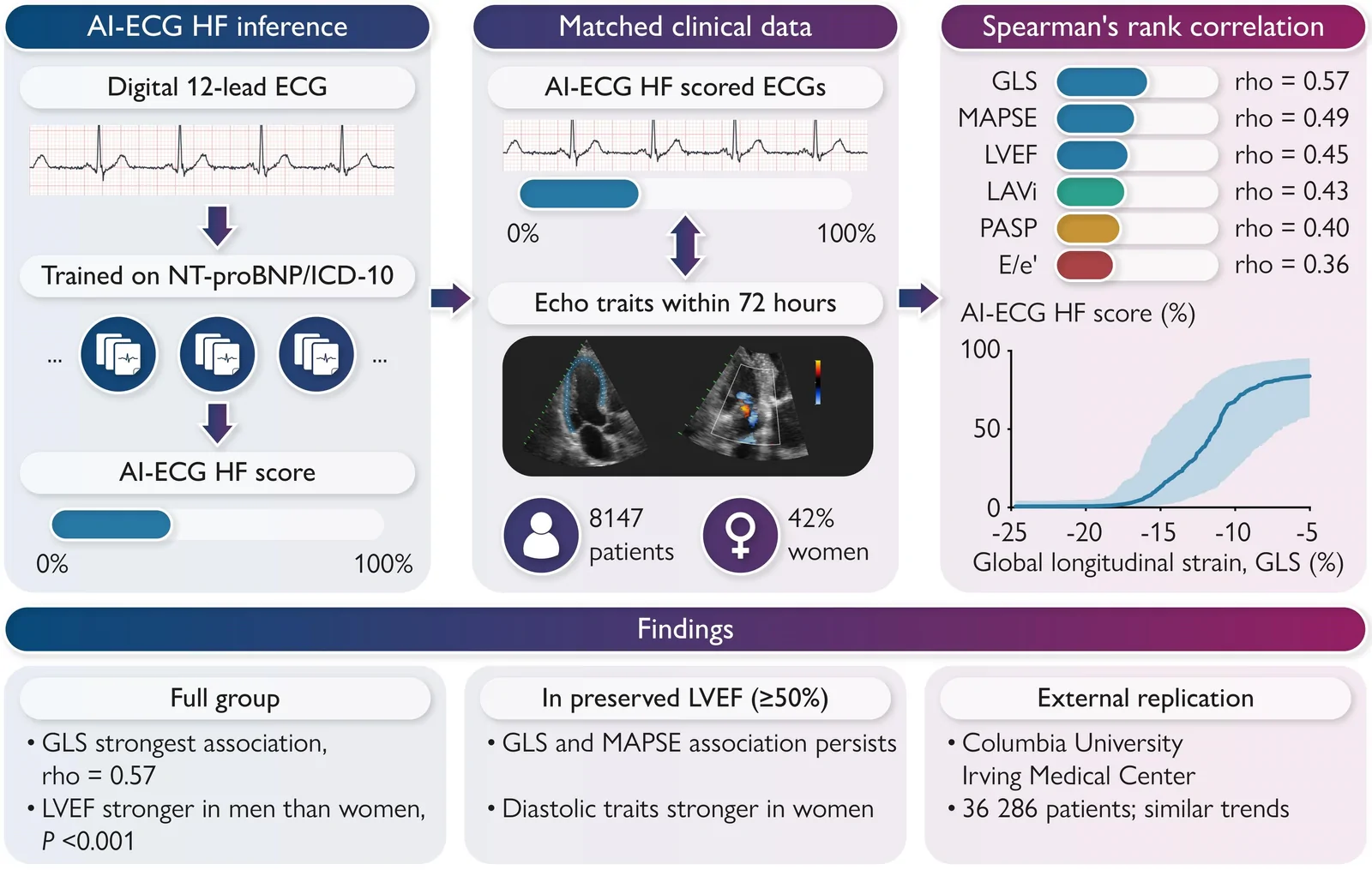

## Introduction

Cardiovascular disease is the leading cause of death globally,^1^ and heart failure (HF) is a common end stage of most cardiovascular disease. HF is severely underdiagnosed due to the relatively high diagnostic complexity and low specificity of symptoms such as fatigue and dyspnoea. Current diagnostic algorithms include risk factors, signs, and levels of N-terminal pro-B-type natriuretic peptide (NT-proBNP) to qualify for echocardiographic examination to rule out HF or determine its phenotype. In the last few years, artificial intelligence-interpreted electrocardiography (AI–ECG) has emerged as a promising candidate to improve diagnostics.^2^ AI–ECG models can detect a range of cardiovascular diseases, including HF^3^ and its associated phenotypes^4–6^ but are often perceived as black-box models by clinicians.

Several *post hoc* explainability techniques have been proposed and used on the ECG, such as Gradient-weighted Class Activation Mapping, Integrated Gradients, and Layer-wise Relevance Propagation.^3,7–10^ While these methods may highlight salient features in the ECG, their reliability is limited,^11^ and they provide little mechanistic insight into cardiac function.

To address this limitation, we investigate the use of established echocardiographic parameters as correlates of AI–ECG HF prediction. This approach enables characterization of the HF-related phenotypes associated with AI–ECG HF prediction, bridging the gap between deep learning predictions and measurable cardiac physiology.

### Echocardiography

Echocardiography is a cornerstone in the evaluation of patients with suspected HF, given its widespread availability and ability to provide a real-time assessment of cardiac structure and function.^12,13^ The indices included in the present study are summarized in *Table 1* and briefly described below.

**Table 1:** Echocardiographic parameters considered in this study and their diagnostic roles

Abbreviation	Full name	Diagnostic role
GLS	Global longitudinal strain	LV systolic function
MAPSE	Mitral annular plane systolic excursion	LV systolic function
LVEF	LV ejection fraction	LV systolic function
TAPSE	Tricuspid annular plane systolic excursion	Right ventricular systolic function
CI	Cardiac index	Global pump performance
*E*/*e′*	*E*/*e′* ratio	Estimate of LV filling pressures
*e′*	Average *e′* velocity	Diastolic function
*E*/*A*	Mitral *E*/*A* ratio	Diastolic function
*A*	Mitral A-wave velocity	Diastolic function
LAVi	Left atrial volume index	Chronic elevation of LV filling pressures/diastolic burden
IVS	Interventricular septal thickness	LV hypertrophy
PWT	Posterior wall thickness	LV hypertrophy
LVMi	LV mass index	Hypertrophy and remodelling assessment
LVEDVi	LV end-diastolic volume index	LV dilation/chamber size assessment
PASP	Pulmonary artery systolic pressure	Pulmonary hypertension

Diagnostic roles follow established echocardiographic interpretation guidelines.^12^

LV, left ventricular.

Left ventricular ejection fraction (LVEF) is calculated as stroke volume divided by end-diastolic volume and remains, despite its well-known limitations, the *lingua franca* for phenotyping HF across populations.^14,15^ HF is conventionally classified into three groups based on LVEF: reduced (≤40%), mildly reduced (41–49%), and preserved (≥50%); this classification accurately identifies advanced systolic HF, but its ability to distinguish cases declines when LVEF is mildly reduced or within the normal range.^16^

Global longitudinal strain (GLS) has, over the past two decades, emerged as a more sensitive and reproducible parameter, capable of detecting myocardial dysfunction even when LVEF is within the normal range, as seen in conditions such as diastolic HF, hypertrophic cardiomyopathy, and amyloidosis.^13,17^ Complementary indices of longitudinal performance are the mitral and tricuspid annular plane systolic excursion (MAPSE/TAPSE), which are obtainable in almost all patients and reflect basal systolic shortening in the left and right ventricles, respectively. For the right ventricle, TAPSE remains the main index for systolic function, with established prognostic value.^12,18^

Diastolic function is assessed using complementary indices reflecting LV relaxation, compliance, and filling pressures, including transmitral inflow, early diastolic mitral annular velocities, and the *E*/*e′* ratio. Pulmonary artery systolic pressure (PASP) and left atrial volume index (LAVi) provide additional markers of filling pressure and diastolic burden, while interventricular septal and posterior wall thickness (IVS/PWT), LV mass, and LV end-diastolic volume index (LVEDVi) characterize structural remodelling.^12,13,16,19^

## Methods

This was a retrospective observational cohort study, reported in accordance with the STROBE guidelines, and approved by the Norwegian Directorate of Health (Helsedirektoratet; case 24/45684-5; dated 24 January 2025). The study included patients admitted to Akershus University Hospital (Ahus) between 1 January 2023 and 1 June 2025. Patients were eligible for inclusion if they had at least one standard 12-lead electrocardiogram (ECG) and one transthoracic echocardiographic (TTE) examination performed within 3 days during the study period. For each patient, the ECG closest in time to the echocardiogram was selected for analysis. Ahus serves as a tertiary care centre within a healthcare network covering approximately 600 000 inhabitants, representing both urban and suburban populations.

Digital ECGs and echocardiograms were retrieved from the hospital’s picture archiving and communication system, where they are stored using the Digital Imaging and Communications in Medicine standard. A previously developed and evaluated AI–ECG model for HF prediction was used to generate an AI–ECG HF prediction score for each ECG.^20^ The model achieved an area under the receiver operating characteristic curve of between 0.86 and 0.96, depending on target definition. The AI–ECG HF prediction score ranges from 0 to 1, with higher values indicating stronger model-estimated signs of HF; for visualization, scores were multiplied by 100 and shown as percentages in figures. The model was trained using a pragmatic label based on International Classification of Diseases, 10th Revision (ICD-10) HF codes and NT-proBNP thresholds, without using echocardiography; positive labels required both coded HF and elevated NT-proBNP. Because of this, the AI–ECG HF prediction score should not be interpreted as a fully calibrated probability of HF. ECGs were preprocessed to the appropriate format for inference, including resampling to 500 Hz and per-lead *z*-normalization.

ICD-10 codes alone were considered insufficient for model training because hospital coding can be incomplete, heterogeneous, and influenced by administrative practice. Adding NT-proBNP was intended to enrich the label for a broader clinical HF syndrome with objective evidence of haemodynamic stress, including presentations where LVEF is preserved. Conversely, training on echocardiographic thresholds would have restricted the target to patients selected for echocardiography and to the particular phenotype chosen, such as reduced ejection fraction, thereby risking selection bias and under-representation of HF with preserved ejection fraction (HFpEF), HF with mildly reduced ejection fraction, or other HF presentations. The model was therefore not designed to detect a single echo-defined phenotype; echocardiographic parameters are used here only to characterize traits associated with the AI–ECG HF prediction score.

Because echocardiographic data were pragmatically collected as part of routine clinical care, only parameters annotated in standard reports were available for analysis. The annotated parameters varied per examination, depending on image quality and clinical indication. A subset of relevant echocardiographic parameters was selected based on relevance within HF diagnostics. Volumes, masses, and cardiac output were indexed to body surface area, estimated using the Du Bois formula,^21^ which is the standard method applied in routine echocardiographic reporting at the hospital.

For external replication of shared echocardiographic associations, the model was evaluated on the EchoNext dataset from Columbia University Irving Medical Center (CUIMC), comprising paired ECG–TTE examinations collected during routine clinical care between 2008 and 2022. Patients were included if an ECG and TTE were performed within 1 year, and only the closest pair per patient was retained, yielding 36286 ECG–TTE pairs from 36 286 patients. These ECGs were resampled to 500 Hz, and per-lead *z*-normalized; the signals were clipped at the 0.1st and 99.9th percentiles. Five echocardiographic parameters were included: visually determined LVEF, PASP, tricuspid regurgitation maximum velocity (TRv), IVS, and PWT. No retraining or calibration of the AI–ECG model was performed.

In both the internal and external test cohorts, pairwise associations between the AI–ECG HF prediction score and each echocardiographic parameter were quantified using Spearman’s rank correlation. Rank correlation was chosen as the variables were expected to be monotonically but not linearly associated. For GLS, and in supplementary parameter-specific analyses for all echocardiographic indices, adjusted partial Spearman correlations were calculated by percentile-rank transforming the AI–ECG HF prediction score and echocardiographic parameters, residualizing them on the adjustment covariates, and correlating the residuals. Models adjusted for age and sex, with or without baseline comorbidities. A GLS-availability sensitivity analysis used stabilized inverse-probability weights based on age, sex, and baseline comorbidities, capped at the 99th percentile, to recalculate the primary GLS correlation. Exploratory internal cohort analyses were stratified by selected machine-detected features on the index ECG (atrial fibrillation/flutter, conduction block, and LV hypertrophy) and by known HF diagnosed before the index ECG. The GLS correlation was also estimated among patients without any of the selected ECG abnormalities and among those without known HF before the index ECG. Confidence intervals (CIs) for unadjusted and adjusted partial Spearman correlations were estimated using the bootstrap percentile method, and significance levels for unadjusted correlations were estimated using permutation testing, each based on 10 000 resamples. To visualize trends, the median AI–ECG HF prediction score was estimated across the distribution of each echocardiographic parameter using kernel smoothing, with shaded bands representing the interquartile range. Statistical analyses were performed in Python 3.12 using numpy 2.2.6, pandas 2.3.0, scipy 1.16.0, and scikit-learn 1.7.0 libraries.

## Results

**Figure 1:**
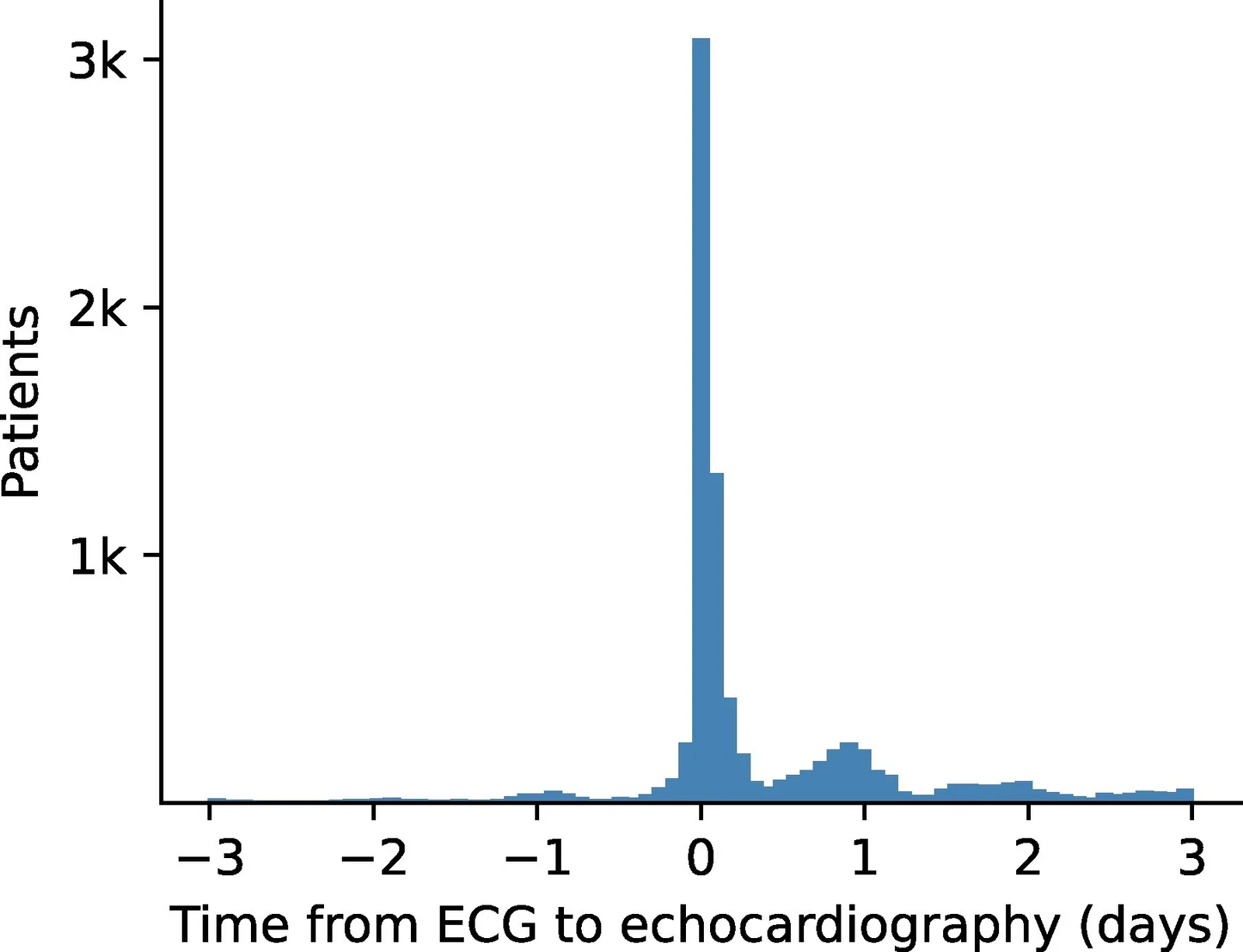
Relative time difference between matched ECG and echocardiographic examination for each patient in the internal Ahus test cohort.

In total, 8147 patients (42% females) with a mean age of 66.4 years were included. The mean absolute time difference between ECG and echocardiogram was 11.2 h, and ECG was usually recorded first. The full distribution of time differences is shown in *Figure 1*. Additional baseline clinical information is presented in *Table 2*. Within the group, 735 (9%) of the patients had been previously diagnosed with HF, and 2293 (28%) diagnosed at any point before data extraction from the electronic health records. Among the 2293 patients with any recorded HF diagnosis, 2007 had an annotated LVEF value in connection to ECG: 587 patients (29%) had LVEF ≤40%, 542 patients (27%) had LVEF in 41–49%, and 878 patients (44%) had LVEF ≥50%.

**Table 2:** Baseline characteristics for the full echocardiography cohort: age, sex, and comorbidities recorded before the index date and across all available time

Characteristic	ICD-10	Before index	All time
Patients, *n*		8147	
Age			
Years, mean (SD)		66.4 (15.6)	
Years, median [IQR]		69 [57 78]	
Sex			
Female		3432 (42%)	
Male		4715 (58%)	
Comorbidities			
Hypertension	I10–I15	2073 (25%)	2966 (36%)
Ischaemic heart disease	I20–I25	1845 (23%)	3149 (39%)
Atrial fibrillation	I48	1320 (16%)	2476 (30%)
Myocardial infarction	I21, I22, I25.2	1078 (13%)	1776 (22%)
Heart failure	I42, I50, I11.0	735 (9%)	2293 (28%)
Chronic obstructive pulmonary disease	J44	530 (7%)	732 (9%)

ICD-10, International Classification of Diseases, 10th Revision; IQR, interquartile range.

Before index includes diagnostic codes registered before the index ECG. All time includes codes recorded at any time in the available EHR, including after the index.

The relationship between the AI–ECG HF prediction score and echocardiographic parameters was assessed using Spearman’s rank correlation, presented in *Table 3*. The most correlated indices, GLS (*ρ* = 0.57, 95% CI 0.54–0.59), MAPSE (*ρ* = −0.49, 95% CI −0.51 to −0.47), and LVEF (*ρ* = −0.45, 95% CI −0.47 to −0.43), are all measures of LV systolic function. They were followed by LAVi, PASP, LV mass index (LVMi), *E*/*e′*, and *e′*. High LAVi and LVMi indicate left atrial dilation and LV hypertrophy, and PASP, *E*/*e′*, and *e′* are related to elevated filling pressures and diastolic function. Finally, TAPSE was inversely correlated with the AI–ECG HF prediction score (*ρ* = −0.34, 95% CI −0.36 to −0.32). The other parameters showed weaker correlations (|*ρ*| ≤ 0.22), suggesting no strong, monotonic relationship within the full patient group.

**Table 3:** Spearman rank correlation between AI–ECG HF score and echocardiographic parameters, calculated on the Ahus cohort

Parameter	*n*	*ρ*	95% CI	
			Low	High
GLS	2899	0.57	0.54	0.59
MAPSE	4177	−0.49	−0.51	−0.47
LVEF	6518	−0.45	−0.47	−0.43
LAVi	4936	0.43	0.41	0.45
PASP	5273	0.40	0.37	0.42
LVMi	4863	0.37	0.35	0.40
*E*/*e′*	4426	0.36	0.34	0.39
*e′*	7203	−0.35	−0.37	−0.33
TAPSE	7248	−0.34	−0.36	−0.32
IVS	2530	0.22	0.19	0.26
PWT	2500	0.22	0.19	0.26
LVEDVi	5179	0.14	0.12	0.17
CI	5175	−0.06	−0.09	−0.03
*A*	6853	0.06	0.03	0.08
*E*/*A*	6846	−0.00	−0.03	0.02

*n* is the number of pairs used for each correlation. Higher absolute *ρ* indicates stronger monotonic association. Values are 95% bootstrap confidence intervals (10 000 resamples).

AI–ECG, artificial intelligence-enabled electrocardiography; CI, cardiac index; CI, confidence interval; HF, heart failure; GLS, global longitudinal strain; IVS, interventricular septal thickness; LAVi, left atrial volume index; LVEF, left ventricular ejection fraction; LVEDVi, left ventricular end-diastolic volume index; LVMi, left ventricular mass index; MAPSE, mitral annular plane systolic excursion; PASP, pulmonary artery systolic pressure; PWT, posterior wall thickness; TAPSE, tricuspid annular plane systolic excursion.

Subgroup analysis based on LVEF and sex was performed and presented in *Table 4*; the crossed LVEF-by-sex analysis is shown in Supplementary material online, *Table S1*. After stratification by LVEF, GLS remained among the most strongly correlated indices. LAVi and PASP also correlated relatively well across the LVEF stratum. Diastolic-related parameters exhibited higher correlations in patients with LVEF ≥50%. There were significant sex differences in LVEF and LVEDVi, with the model being more strongly correlated in men. Conversely, the model was significantly more correlated with *E*/*e′*, PASP, IVS, PWT, CI, and A-wave velocity in women than in men.

**Table 4:** Spearman rank correlation between parameters and AI–ECG HF score stratified by LVEF subgroup and sex, calculated on the Ahus cohort

	LVEF subgroup			Sex		
	≤40%	41–49%	≥50%	Male	Female	*P*
GLS	0.44	0.34	0.37	0.57	0.54	0.252
MAPSE	−0.25	−0.30	−0.35	−0.48	−0.52	0.185
LVEF	−0.26	−0.20	−0.16	−0.49	−0.39	<0.001
LAVi	0.32	0.33	0.36	0.41	0.44	0.265
PASP	0.26	0.29	0.37	0.38	0.43	0.027
LVMi	0.21	0.18	0.28	0.35	0.40	0.070
*E/e′*	0.25	0.24	0.36	0.32	0.45	<0.001
*e′*	−0.20	−0.18	−0.31	−0.36	−0.36	0.875
TAPSE	−0.29	−0.21	−0.22	−0.33	−0.36	0.175
IVS	−0.05	0.12	0.26	0.11	0.34	<0.001
PWT	0.05	0.09	0.26	0.11	0.33	<0.001
LVEDVi	0.22	0.04	−0.02	0.22	−0.01	<0.001
CI	0.06	0.01	−0.02	−0.03	−0.12	0.001
*A*	−0.11	−0.02	0.13	0.03	0.12	<0.001
*E/A*	0.14	0.15	−0.06	0.02	−0.03	0.075

Higher absolute *ρ* indicates stronger monotonic association. The *P* column reports two-sided permutation test *P*-values for the difference in correlation between males and females, based on 10 000 random reassignments of sex labels. Values are capped at <0.001.

AI–ECG, artificial intelligence-enabled electrocardiography; CI, cardiac index; CI, confidence interval; HF, heart failure; GLS, global longitudinal strain; IVS, interventricular septal thickness; LAVi, left atrial volume index; LVEF, left ventricular ejection fraction; LVEDVi, left ventricular end-diastolic volume index; LVMi, left ventricular mass index; MAPSE, mitral annular plane systolic excursion; PASP, pulmonary artery systolic pressure; PWT, posterior wall thickness; TAPSE, tricuspid annular plane systolic excursion.

The association between GLS and the AI–ECG HF prediction score persisted after adjustment for age and sex using partial Spearman rank correlation (partial *ρ* = 0.51, 95% CI 0.48–0.54; *Table 5*). The association also remained present after additional adjustment for baseline comorbidities (partial *ρ* = 0.49, 95% CI 0.46–0.52). Across parameter-specific adjusted analyses including all echocardiographic indices, GLS retained the largest absolute partial correlation after adjustment for age and sex and after additional adjustment for baseline comorbidities (see Supplementary material online, *Tables S4* and *S5*). An extensive GLS-availability comparison is shown in Supplementary material online, *Table S2*. An inverse probability-weighted sensitivity analysis for GLS availability produced an estimate similar to the primary analysis (see Supplementary material online, *Table S3*).

**Table 5:** Adjusted partial Spearman rank correlations between GLS and the AI–ECG HF prediction score

Adjustment set	*n*	Partial *ρ*	95% CI		*P*
			Low	High	
Age and sex	2899	0.51	0.48	0.54	<0.001
+ Comorbidities	2899	0.49	0.46	0.52	<0.001

Partial *ρ* is the correlation between ranked AI–ECG and ranked GLS residuals after adjustment. ‘+ Comorbidities’ additionally adjusts for baseline atrial fibrillation, chronic obstructive pulmonary disease, HF, hypertension, ischaemic heart disease, and myocardial infarction.

AI–ECG, artificial intelligence-enabled electrocardiography; HF, heart failure; GLS, global longitudinal strain.

**Figure 2:**
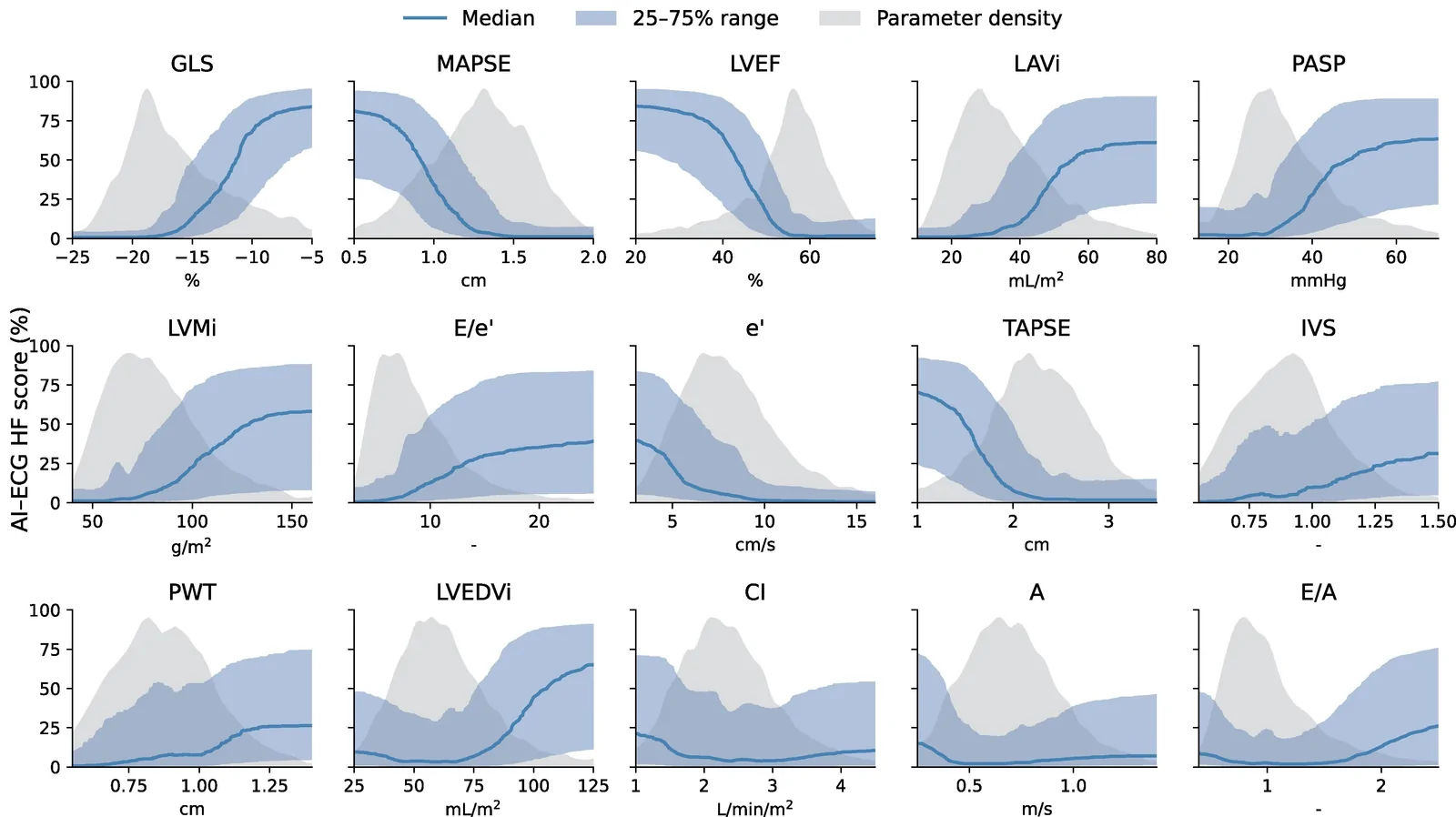
Median and interquartile range of the AI–ECG HF prediction score as a function of varying echocardiographic parameters in the internal evaluation dataset, sorted by Spearman’s rank correlation.

We plotted the kernel-estimated median of the AI–ECG HF prediction score as a function of each parameter, along with the interquartile range of the data, in *Figure 2*. We found that the least correlated parameters often exhibited non-monotonic relationships, where the AI–ECG HF prediction score increased at both extremes.

**Figure 3:**
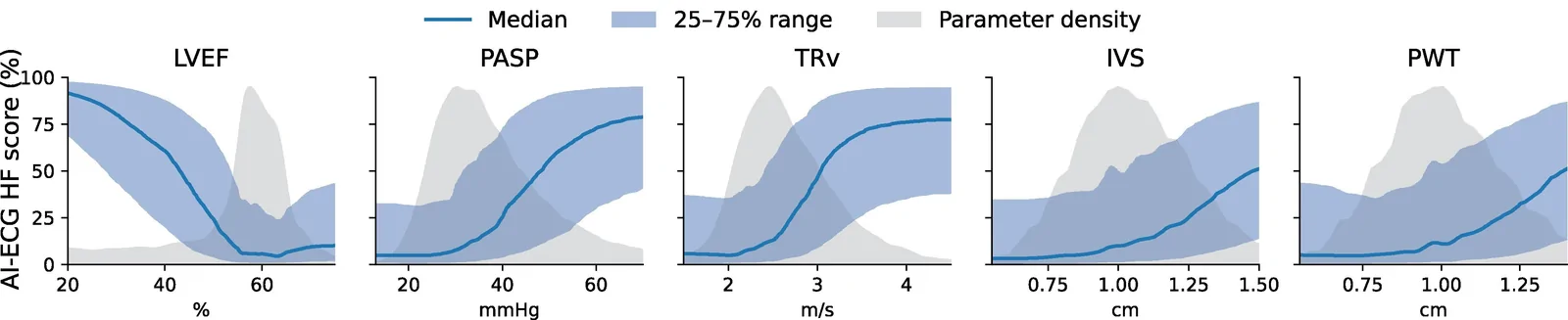
Median and interquartile range of the AI–ECG HF prediction score as a function of varying echocardiographic parameters from the EchoNext dataset, collected at Columbia University Irving Medical Center.

The same kernel-smoothing procedure was applied to the external EchoNext dataset, shown in *Figure 3*, which consisted of 36 286 ECG–TTE pairs. Compared with the internal cohort, the external dataset exhibited a higher median AI–ECG HF prediction score across the full range of echocardiographic parameters. Despite these shifts, the qualitative relationships between AI–ECG HF prediction and available echocardiographic traits remained similar.

**Figure 4:**
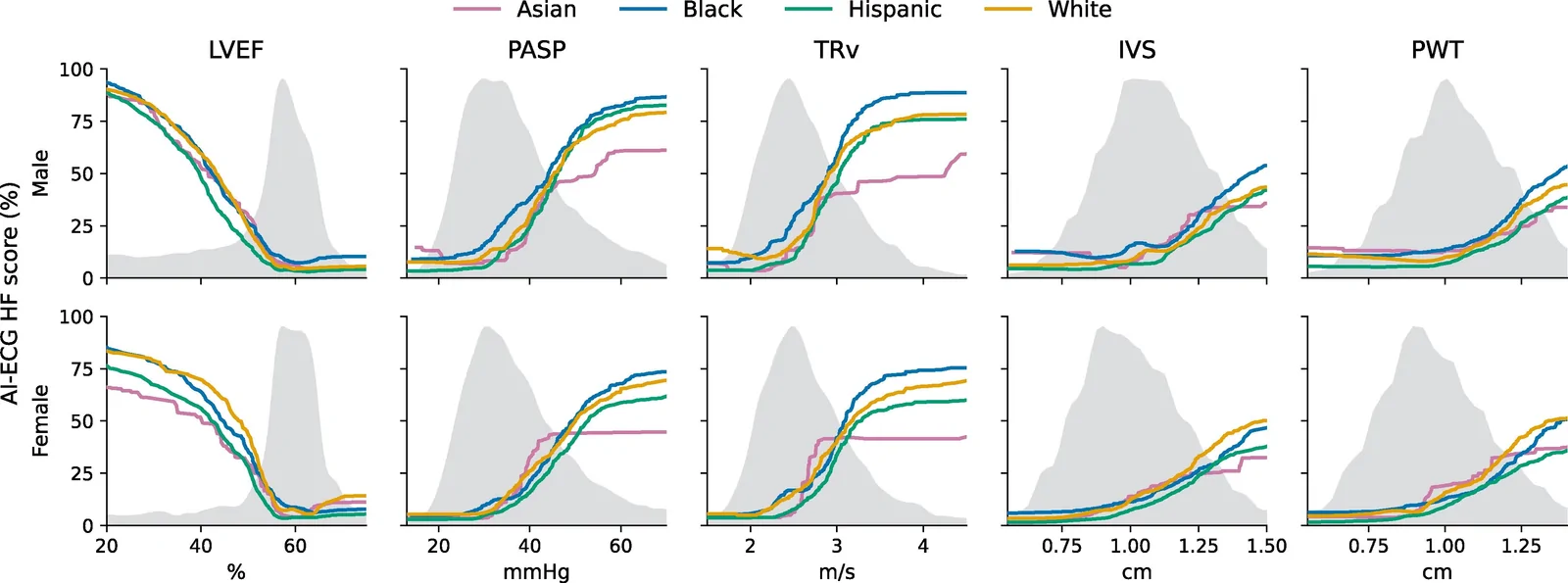
Median AI–ECG HF prediction score stratified by echocardiographic parameters, sex, and race, from the EchoNext dataset, collected at CUIMC.

**Figure 5:**
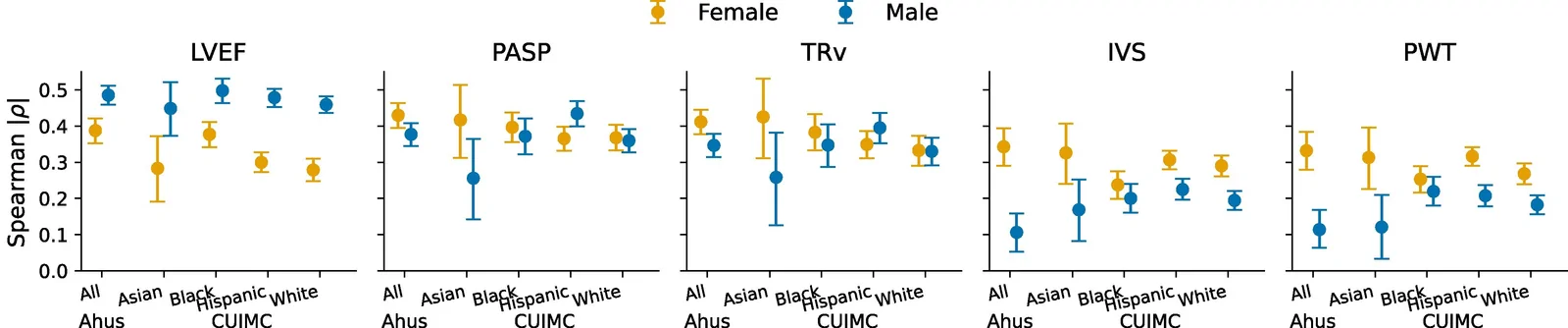
Absolute Spearman’s rank correlation (|*ρ*|) with 95% bootstrap percentile confidence intervals for five echocardiographic indices, split by medical site, sex, and race.

In the external CUIMC cohort, race was reported for each ECG–TTE pair, enabling stratification by sex and race. The kernel-based median as a function of echocardiographic indices is shown in *Figure 4*, with corresponding group sizes presented in *Table 6*. Black men were generally assigned a higher AI–ECG HF prediction score, and Asian men were assigned relatively lower scores at abnormal echocardiographic values. It should, however, be noted that the Asian group was by far the smallest. To assess differences between the Ahus cohort and CUIMC, rank correlations of the common echocardiographic indices are presented in *Figure 5*. The AI–ECG HF prediction score correlated more strongly with LVEF in men than in women across all groups. For IVS and PWT, the same directional relationship was seen in the CUIMC cohort as reported in *Table 4*, but the sex differences were generally smaller.

**Table 6:** Counts of patients by race and sex

Race	Total	Male	Female
Hispanic	11 006	4782	6224
White	10 635	6037	4598
Black	5424	2408	3016
Unknown	5159	2635	2524
Other	2980	1530	1450
Asian	1082	561	521

The full correlation matrix of all parameters sorted by absolute correlation with the AI–ECG HF prediction score is displayed in Supplementary material online, *Figure S1*. Exploratory subgroup analyses in the Ahus cohort are shown in Supplementary material online, *Figures S2* and *S3*. Patients with atrial fibrillation/flutter recorded on the selected ECG generally had higher AI–ECG HF prediction scores across the echocardiographic ranges, as did patients with known HF before the index ECG. However, the GLS association remained similar among patients without any of the selected machine-detected ECG abnormalities (*ρ* = 0.54, 95% CI 0.50–0.57; *n* = 2337) and among patients without known HF before the index ECG (*ρ* = 0.53, 95% CI 0.50–0.56; *n* = 2617), compared with the full-cohort association (*ρ* = 0.57, 95% CI 0.54–0.59; *n* = 2899; Supplementary material online, *Table S6*).

## Discussion

Overall, GLS showed the strongest association, followed by MAPSE and LVEF; the LVEF association was stronger in men than in women. Among patients with LVEF ≥50%, the score remained associated with GLS and MAPSE, as well as with diastolic and remodelling markers, including LAV, pulmonary pressures, and the *E*/*e′* ratio. Sex-stratified analyses showed stronger associations in women for several diastolic, pulmonary pressure, and wall thickness indices, whereas LVEF and LVEDVi were more strongly associated in men.

The relatively weaker correlation between AI–ECG HF prediction score and LVEF than between AI–ECG HF prediction score and GLS suggests that the score is associated with systolic impairment that is not captured by volumetric measures alone. About half of HF patients have preserved LVEF, and systolic dysfunction in these patients is still measurable via GLS,^22^ possibly explaining its higher correlation. The persistence of the GLS association after adjustment, and its similar magnitude among patients without known HF before the index ECG or the selected machine-detected ECG abnormalities, suggests that this relationship is not explained solely by age, sex, recorded baseline comorbidity, established HF, or these ECG abnormalities. The associations to diastolic and filling pressure-related parameters were most pronounced in patients with preserved LVEF, indicating that AI–ECG HF prediction is associated with echocardiographic traits relevant to HFpEF, without establishing that the model specifically identifies clinical HFpEF.

A notable result was the difference in associations between men and women. In the crossed LVEF-by-sex analysis, several diastolic, pulmonary pressure, and wall thickness indices were more strongly associated with the score in women with LVEF ≥50% (see Supplementary material online, *Table S1*). These sex-stratified findings should be considered hypothesis-generating. Several non-exclusive explanations may contribute, including sex differences in HFpEF phenotypes, body size and ventricular remodelling patterns, ECG expression of myocardial or loading abnormalities, referral patterns, and echocardiographic measurement practices. Because the AI–ECG model was trained on pragmatic clinical labels, sex-related differences in diagnostic coding, NT-proBNP distributions, comorbidity burden, or clinical workflows may also influence the observed associations. The present analysis cannot determine whether the model uses sex-specific ECG features directly or whether these differences reflect correlated clinical context.

The AI–ECG HF prediction score increased sharply near clinically relevant thresholds, which aligns with how systolic and diastolic dysfunction is interpreted in routine practice. Patients with GLS more negative than approximately −18% consistently had low AI–ECG HF prediction scores, whereas those with GLS less negative than approximately −16% had increasingly higher scores. Patients with LVEF of above 55% also had low scores, whereas patients with LVEF ≤40% had consistently high scores. The smoothed AI–ECG score increased progressively at PASP values above approximately 30 mmHg.

Evaluation on an external EchoNext dataset collected at CUIMC showed similar relationship patterns, but with notable differences. The distribution of echocardiographic indices differed, with a much larger proportion of patients having very low LVEF. Differences likely reflect variations in patient demographics, acquisition hardware, and echocardiographic reporting; LVEF was visually estimated in the external cohort, and ECG–TTE pairs were allowed within one year rather than three days. The AI–ECG HF prediction score was generally higher in the external cohort, but its correlation with available parameters was similar. The consistency supports external replication of shared echocardiographic associations but does not reproduce the full internal echocardiographic phenotype analysis. Stratified by race and sex, the highest median AI–ECG HF prediction scores were assigned to Black males and the lowest to Asian men. The results may reflect differences in HF prevalence between groups, but this cannot be verified because diagnostic labels are not included in the CUIMC dataset.

Unlike AI–ECG models trained to detect specific echo-defined targets such as reduced EF or diastolic dysfunction, the model evaluated here was trained to detect hospital-diagnosed HF supported by NT-proBNP thresholds. The present results should therefore be interpreted as echocardiographic trait characterization of a broad HF prediction model, not as evidence that the model directly detects individual echocardiographic parameters.

Current explainability methods for AI–ECG fall into two categories: inherent and *post hoc*, each with limitations. Inherent methods, such as variational autoencoding-based models, offer human-interpretable latent features but cause substantial performance drops compared with less interpretable models.^3,23^  *Post hoc* approaches avoid this performance drop but introduce other issues: instance-level explanations can increase human agreement even with incorrect AI outputs, thus reinforcing confirmation bias,^24–26^ and despite fostering trust, clinical trials have not demonstrated that explainable systems improve outcomes relative to black-box models.^27^  *Post hoc* methods can nevertheless be useful in specific scenarios; when models operate on paper-ECG images, saliency maps may highlight regions unrelated to the task and thereby provide partial insight. In contrast, interpreting saliency maps on digital ECGs is challenging. High saliency on any given signal segment may reflect physiological features or undesired confounding measurement noise, with no clear way to disentangle them.

Cohort-level correlation analysis offers a practical and clinically intuitive way to interpret AI–ECG models. Identifying the echocardiographic traits most strongly associated with AI–ECG HF prediction may help contextualize model behaviour, reveal subgroups in which the model behaves differently, and guide targeted improvements in model development. The observed associations with GLS and diastolic-related traits may inform future studies of echocardiography prioritization in selected patients, such as those with unexplained dyspnoea. A related exploratory use case is high-risk groups in whom GLS monitoring is already clinically relevant, such as patients receiving potentially cardiotoxic therapy. These possible applications were not tested in the present study and require prospective evaluation.

The present study should be interpreted with its limitations in mind. First, the AI–ECG model was trained using pragmatic clinical labels rather than adjudicated HF labels. Its outputs may therefore reflect diagnostic coding, NT-proBNP testing, clinical workflows, and cardiac physiology. Because NT-proBNP contributed to the training label and is biologically related to ventricular dysfunction, filling pressures, and cardiac remodelling, associations between the AI–ECG HF prediction score and echocardiographic abnormalities are partly expected. The present analysis should therefore be interpreted as echocardiographic trait characterization of the model output, rather than independent physiological validation. Second, subgroup interpretations, including the sex-stratified and preserved-LVEF findings discussed above, should be considered exploratory and cannot establish specific HFpEF-related or sex-specific model mechanisms. Third, time differences between ECG and echocardiographic examinations may have attenuated the observed correlations: in the Ahus cohort, the interval was always less than 3 days, whereas the external cohort allowed ECG–TTE pairs within 1 year and the exact time interval was not available, precluding shorter-window sensitivity analyses. Underestimation of associations is therefore more likely in the external cohort. Fourth, echocardiographic missingness was partly driven by clinical indication, measurement practice, and image quality, and is therefore unlikely to be random for several clinically relevant parameters. Finally, cohort-level explanations do not capture all the factors that drive individual predictions, and individual failure modes will not necessarily be found using the method.

An AI–ECG model trained to detect hospital-diagnosed HF supported by NT-proBNP thresholds was associated with established echocardiographic markers of systolic and diastolic dysfunction. This cohort-level interpretability approach provides insight into echocardiographic traits associated with AI–ECG HF prediction and highlights meaningful subgroup differences. Future work should test whether such trait-based interpretation improves model development, clinical interpretation, or prospective use.

## Supplementary Material

ztag141_Supplementary_Data

## Data Availability

The analysis code is available at github.com/Ahus-AIM/Associations-Between-Echocardiographic-Traits-and-AI-ECG-Predictions-of-Heart-Failure. Individual-level clinical data are not publicly available because they contain sensitive health information from routine clinical care and are subject to institutional and legal restrictions.
