# Supplementary material for: Associations between echocardiographic traits and artificial intelligence-enabled electrocardiography predictions of heart failure
Source: Eur Heart J Digit Health. 2026 Sep 11;7(8):ztag141. doi: 10.1093/ehjdh/ztag141 (PMC13584927; doi:10.1093/ehjdh/ztag141)
Supplement: Supplementary file 1 [file ztag141_supplementary_data.pdf]

Supplementary Table 1: Spearman rank correlation between parameters and AI–ECG HF score stratified by both LVEF subgroup and sex, calculated on the Ahus cohort.

	LVEF $\leq$ 40%			LVEF 41–49%			LVEF $\geq$ 50%		
Parameter	Male	Female	p	Male	Female	p	Male	Female	p
GLS	0.44	0.44	0.996	0.35	0.33	0.790	0.34	0.38	0.393
MAPSE	−0.22	−0.31	0.392	−0.30	−0.26	0.694	−0.31	−0.40	0.007
LVEF	−0.34	−0.08	0.002	−0.23	−0.13	0.127	−0.17	−0.15	0.594
LAVi	0.29	0.41	0.180	0.33	0.31	0.721	0.35	0.37	0.570
PASP	0.34	0.08	0.003	0.29	0.27	0.769	0.33	0.41	0.005
LVMi	0.23	0.24	0.919	0.20	0.20	0.983	0.25	0.32	0.026
E/e'	0.22	0.30	0.479	0.26	0.10	0.060	0.31	0.43	<0.001
e'	−0.26	−0.06	0.022	−0.22	−0.04	0.008	−0.28	−0.34	0.025
TAPSE	−0.31	−0.26	0.592	−0.15	−0.29	0.048	−0.20	−0.25	0.060
IVS	−0.08	0.07	0.318	0.12	0.18	0.598	0.15	0.36	<0.001
PWT	0.00	0.13	0.364	0.07	0.27	0.094	0.18	0.34	<0.001
LVEDVi	0.23	0.23	0.987	0.11	−0.03	0.061	0.06	−0.14	<0.001
CI	0.04	0.11	0.532	0.04	−0.02	0.405	0.04	−0.10	<0.001
A	−0.07	−0.17	0.287	−0.01	−0.06	0.500	0.11	0.17	0.051
E/A	0.10	0.23	0.171	0.15	0.16	0.848	−0.05	−0.07	0.491

Higher absolute ρ indicates stronger monotonic association. Within each LVEF subgroup, the p columns report two-sided permutation test p-values for the difference in correlation between males and females.

Supplementary Table 2: Baseline characteristics stratified by GLS availability.

Characteristic	GLS present	GLS missing	p
N	2899	5248	
age	64.6 ± 15.1	67.5 ± 15.8	< 0.001
Male sex	1641 (57%)	3074 (59%)	0.087
Hypertension	658 (23%)	1415 (27%)	< 0.001
COPD	151 (5%)	379 (7%)	< 0.001
Ischaemic heart disease	677 (23%)	1168 (22%)	0.257
Atrial fibrillation	365 (13%)	955 (18%)	< 0.001
Heart failure	282 (10%)	453 (9%)	0.106
Myocardial infarction	411 (14%)	667 (13%)	0.065

Continuous variables are mean ± SD. Binary variables are shown as n (%). Comorbidities were identified from diagnoses preceding the index ECG. The p column uses Welch's t-test for continuous variables and Fisher's exact or chi-square tests for discrete variables.

Supplementary Table 3: Inverse-probability-weighted sensitivity analysis for GLS availability.

Analysis	n	Complete-case ρ	IPW ρ	IPW 95% CI
GLS Spearman correlation	2,899	0.57	0.57	0.54–0.60

IPW denotes stabilised inverse-probability weighting. GLS availability was modelled using age, sex, and pre-index diagnoses of heart failure, atrial fibrillation, hypertension, and ischaemic heart disease. Weights were capped at the 99th percentile (maximum weight 1.57). Confidence intervals are bootstrap percentile intervals for the IPW estimate from 1,000 resamples with refitting of the GLS-availability model.

Supplementary Table 4: Parameter-specific partial Spearman rank correlations adjusted for age and sex.

Parameter	n	Unadjusted ρ	Partial ρ	Partial ρ 95% CI	
				Low	High
GLS	2,899	0.57	0.51	0.48	0.54
MAPSE	4,177	−0.49	−0.42	−0.45	−0.39
LVEF	6,518	−0.45	−0.43	−0.45	−0.41
LAVi	4,936	0.43	0.32	0.29	0.35
PASP	5,273	0.40	0.30	0.28	0.33
LVMi	4,863	0.37	0.30	0.27	0.33
E/e'	4,426	0.36	0.25	0.22	0.28
e'	7,203	−0.35	−0.21	−0.23	−0.18
TAPSE	7,248	−0.34	−0.29	−0.31	−0.27
IVS	2,530	0.22	0.09	0.05	0.12
PWT	2,500	0.22	0.09	0.05	0.13
LVEDVi	5,179	0.14	0.20	0.17	0.22
CI	5,175	−0.06	0.01	−0.02	0.04
A	6,853	0.06	−0.08	−0.11	−0.06
E/A	6,846	0.00	0.14	0.11	0.16

Each row uses the complete-case sample for that echocardiographic parameter and adjustment set. Where included, comorbidities were identified from diagnoses preceding the index ECG. Rows follow the same order as the primary unadjusted Spearman correlation table and are not sorted by adjusted association strength. Confidence intervals are bootstrap percentile intervals for partial ρ from 10,000 resamples.

Supplementary Table 5: Parameter-specific partial Spearman rank correlations adjusted for age, sex, and baseline atrial fibrillation, chronic obstructive pulmonary disease, heart failure, hypertension, ischaemic heart disease, and myocardial infarction.

Parameter	n	Unadjusted ρ	Partial ρ	Partial ρ 95% CI	
				Low	High
GLS	2,899	0.57	0.49	0.46	0.52
MAPSE	4,177	−0.49	−0.40	−0.42	−0.37
LVEF	6,518	−0.45	−0.41	−0.44	−0.39
LAVi	4,936	0.43	0.29	0.26	0.32
PASP	5,273	0.40	0.30	0.27	0.32
LVMi	4,863	0.37	0.28	0.25	0.31
E/e'	4,426	0.36	0.25	0.22	0.28
e'	7,203	−0.35	−0.22	−0.24	−0.19
TAPSE	7,248	−0.34	−0.26	−0.29	−0.24
IVS	2,530	0.22	0.08	0.04	0.12
PWT	2,500	0.22	0.09	0.05	0.13
LVEDVi	5,179	0.14	0.19	0.16	0.21
CI	5,175	−0.06	0.02	−0.01	0.05
A	6,853	0.06	−0.07	−0.09	−0.04
E/A	6,846	0.00	0.12	0.09	0.14

Each row uses the complete-case sample for that echocardiographic parameter and adjustment set. Where included, comorbidities were identified from diagnoses preceding the index ECG. Rows follow the same order as the primary unadjusted Spearman correlation table and are not sorted by adjusted association strength. Confidence intervals are bootstrap percentile intervals for partial ρ from 10,000 resamples.

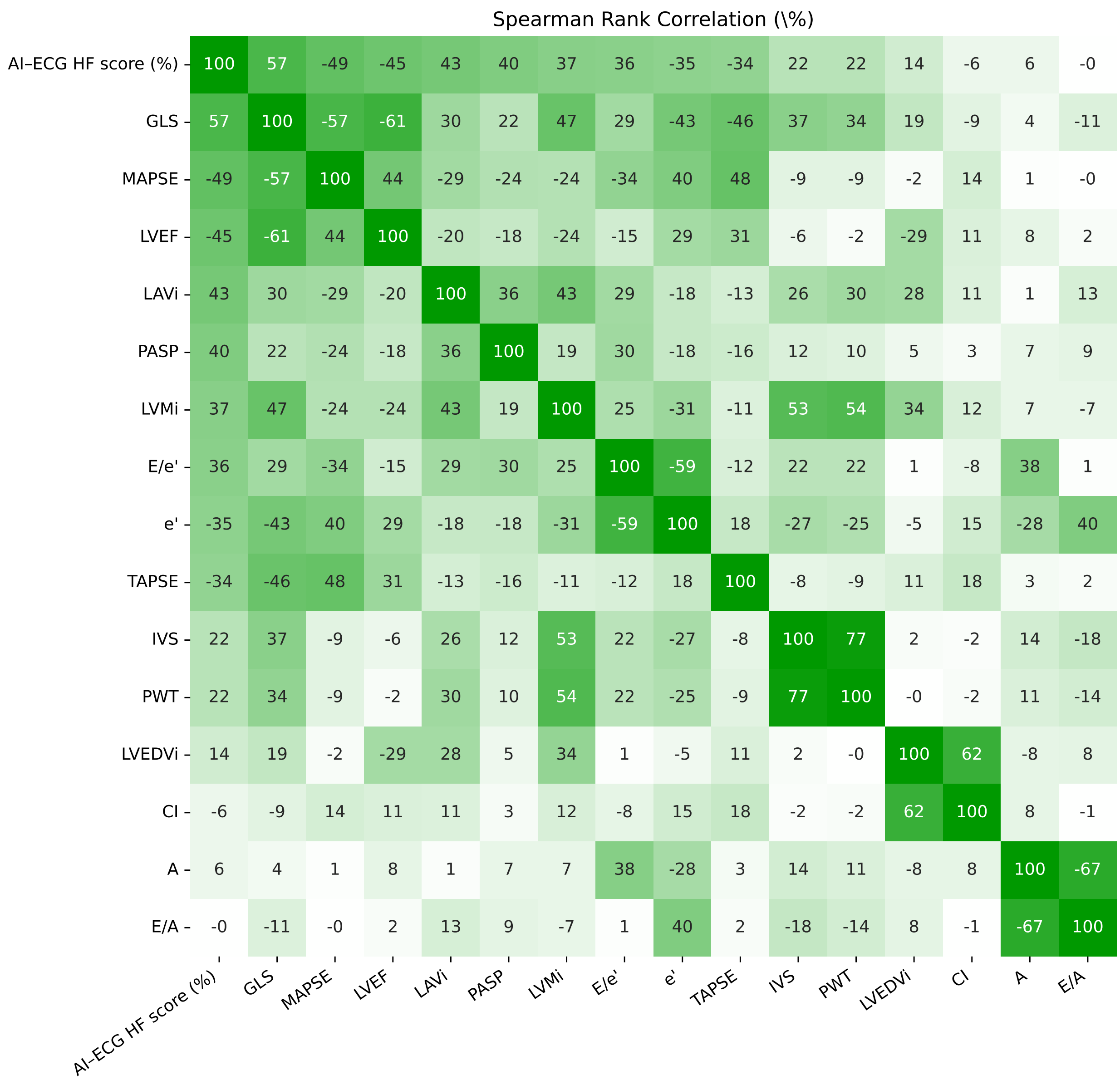

Supplementary Figure 1: Spearman rank correlation across all considered echocardiographic parameters, including the AI–ECG HF prediction score.
Alt text: Correlation matrix heat map showing pairwise Spearman correlations among echocardiographic parameters and the AI–ECG HF prediction score.

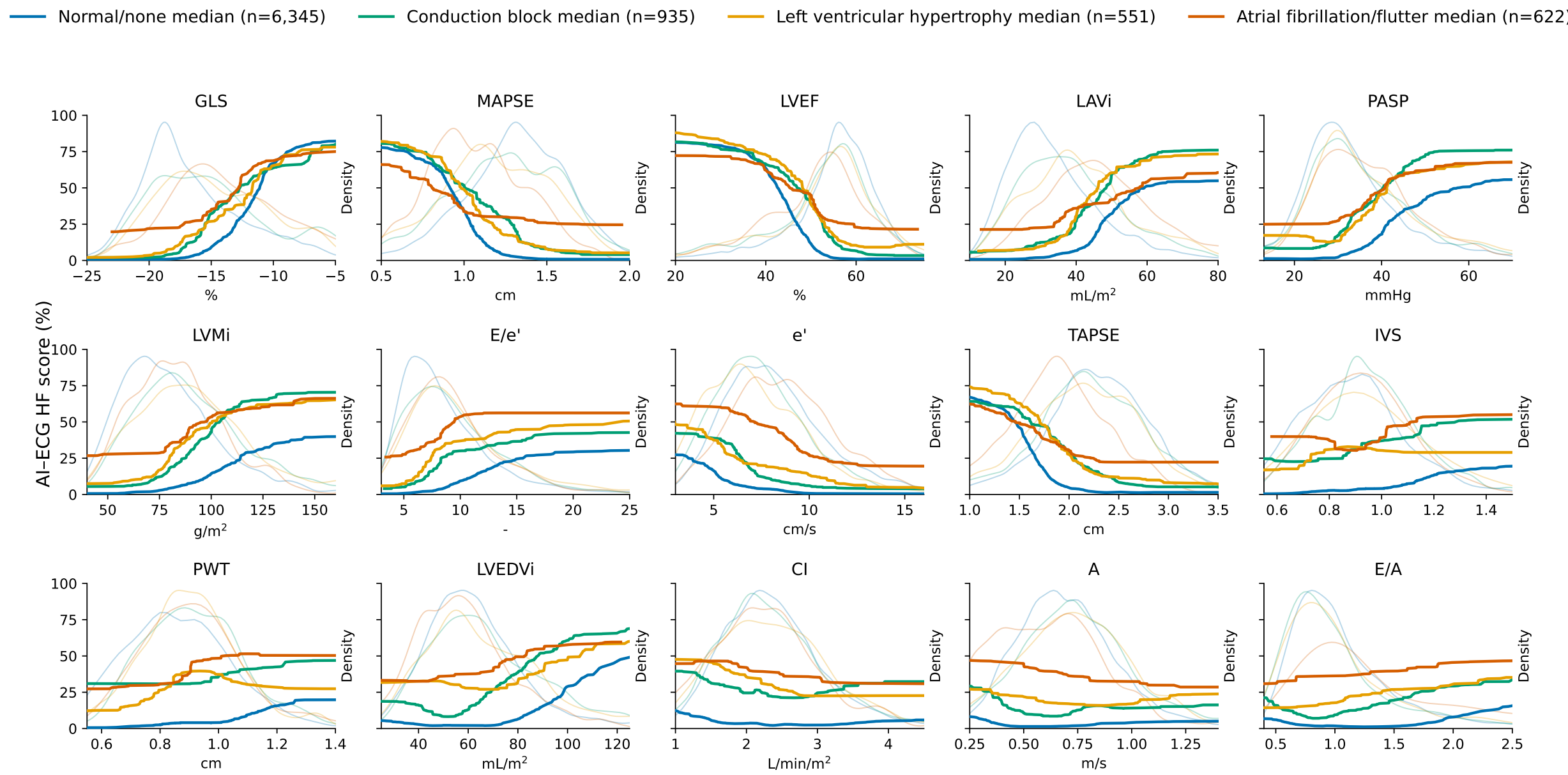

Supplementary Figure 2: Kernel-smoothed median AI–ECG HF prediction score across echocardiographic parameters, stratified by machine-measured ECG features from the selected ECG. Atrial fibrillation/flutter, conduction block, and left ventricular hypertrophy were treated as non-exclusive feature groups, and ECGs without any of these features were grouped as normal/none. Density curves show the echocardiographic parameter distribution within each ECG-feature group.
Alt text: Multi-panel plots showing median AI–ECG HF prediction scores across echocardiographic parameters, stratified by ECG feature groups including atrial fibrillation/flutter, conduction block, left ventricular hypertrophy, and normal/none.

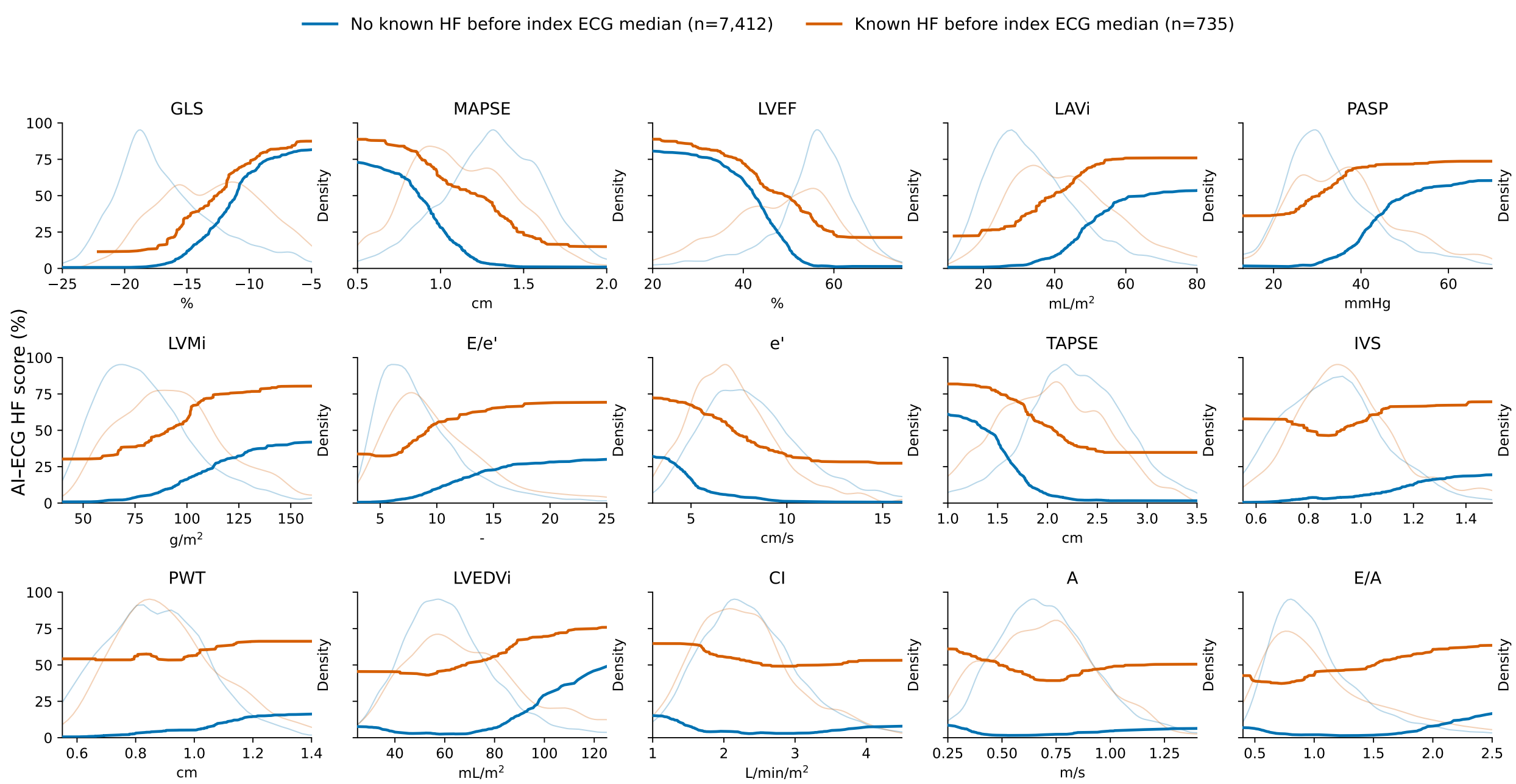

Supplementary Figure 3: Kernel-smoothed median AI–ECG HF prediction score across echocardiographic parameters, stratified by known heart failure before the index ECG, extracted via International Classification of Diseases, 10th Revision codes. Density curves show the echocardiographic parameter distribution among patients with and without known heart failure before the index ECG.
Alt text: Multi-panel plots comparing AI–ECG HF prediction scores across echocardiographic parameter ranges in patients with and without known heart failure before the index ECG.

Supplementary Table 6: Spearman rank correlation between GLS and the AI–ECG HF prediction score in the full cohort and sensitivity-analysis groups.

Group	n	ρ	95% CI
Full cohort	2,899	0.57	0.54–0.59
No selected machine-detected ECG abnormality	2,337	0.54	0.50–0.57
No known HF before index ECG	2,617	0.53	0.50–0.56

n is the number of patients with available GLS. Confidence intervals were estimated using the bootstrap percentile method with 10,000 resamples. Selected machine-detected ECG abnormalities were atrial fibrillation/flutter, conduction block, and left ventricular hypertrophy on the index ECG. Known HF before the index ECG was identified from preceding International Classification of Diseases, 10th Revision codes.
